# Hyperspectral evaluation of hepatic oxygenation in a model of total vs. arterial liver ischaemia

**DOI:** 10.1038/s41598-020-72915-6

**Published:** 2020-09-22

**Authors:** Eric Felli, Mahdi Al-Taher, Toby Collins, Andrea Baiocchini, Emanuele Felli, Manuel Barberio, Giuseppe Maria Ettorre, Didier Mutter, Veronique Lindner, Alexandre Hostettler, Sylvain Gioux, Catherine Schuster, Jacques Marescaux, Michele Diana

**Affiliations:** 1 Institute of Physiology, EA3072 Mitochondria Respiration and Oxidative Stress, University of Strasbourg, Strasbourg, France; 2 IHU-Strasbourg, Institute of Image-Guided Surgery, Strasbourg, France; 3 Department of General, Digestive, and Endocrine Surgery, University Hospital of Strasbourg, Strasbourg, France; 4 INSERM, Institute of Viral and Liver Disease, U1110 Strasbourg, France; 5 Department of Transplantation and General Surgery, San Camillo Hospital, Rome, Italy; 6 Department of Pathology, San Camillo Forlanini Hospital, Rome, Italy; 7 Surgical Data Science Department, Research Institute Against Digestive Cancer (IRCAD), Strasbourg, France; 8 Department of Visceral, Transplant, Thoracic and Vascular Surgery, University Hospital of Leipzig, Leipzig, Germany; 9 Department of Pathology, University Hospital, Strasbourg, France; 10 ICUBE Laboratory, Photonics Instrumentation for Health, University of Strasbourg, Strasbourg, France; 11 University of Strasbourg, Strasbourg, France

**Keywords:** Translational research, Hepatology

## Abstract

Liver ischaemia reperfusion injury (IRI) is a dreaded pathophysiological complication which may lead to an impaired liver function. The level of oxygen hypoperfusion affects the level of cellular damage during the reperfusion phase. Consequently, intraoperative localisation and quantification of oxygen impairment would help in the early detection of liver ischaemia. To date, there is no real-time, non-invasive, and intraoperative tool which can compute an organ oxygenation map, quantify and discriminate different types of vascular occlusions intraoperatively. Hyperspectral imaging (HSI) is a non-invasive optical methodology which can quantify tissue oxygenation and which has recently been applied to the medical field. A hyperspectral camera detects the relative reflectance of a tissue in the range of 500 to 1000 nm, allowing the quantification of organic compounds such as oxygenated and deoxygenated haemoglobin at different depths. Here, we show the first comparative study of liver oxygenation by means of HSI quantification in a model of total vascular inflow occlusion (VIO) vs. hepatic artery occlusion (HAO), correlating optical properties with capillary lactate and histopathological evaluation. We found that liver HSI could discriminate between VIO and HAO. These results were confirmed via cross-validation of HSI which detected and quantified intestinal congestion in VIO. A significant correlation between the near-infrared spectra and capillary lactate was found (r = − 0.8645, p = 0.0003 VIO, r = − 0.7113, p = 0.0120 HAO). Finally, a statistically significant negative correlation was found between the histology score and the near-infrared parameter index (NIR) (r = − 0.88, p = 0.004). We infer that HSI, by predicting capillary lactates and the histopathological score, would be a suitable non-invasive tool for intraoperative liver perfusion assessment.

## Introduction

Liver ischaemia and reperfusion injury (IRI) is a dreaded vascular complication characterised by the disruption of parenchymal and microvascular architecture which leads to hepatic functional impairment^1^. IRI has practical relevance in liver transplantation and during liver surgery performed with intermittent vascular inflow occlusion. Liver oxygenation impairment and ischaemia can be challenging to detect intraoperatively, which is partly due to multiple hepatic vascular inflows^1–3^. Parenchymal disruption in the reperfusion phase mainly depends on the ischaemic time duration. Consequently, intraoperative localisation and quantification of oxygen impairment may be helpful to quickly detect future reperfusion injury sites. To date, there is no tool which can spatially visualise and quantify liver oxygenation intraoperatively.

Currently, hepatic circulation can be evaluated intraoperatively using ultrasound (US). However, US may be time-consuming, especially during laparoscopic surgical procedures, and has a long learning curve^4^. Additionally, US evaluation might be difficult in obese patients and the interpretation is strongly operator-dependent^5^. Besides, US aims to analyse and quantify blood circulation in a specific area of interest and does not provide an immediate localisation and quantification of oxygenation of the whole liver surface.

Hyperspectral imaging (HSI) is a non-invasive technique which has been recently applied to the medical field as a tool for image-guided surgery and specifically for an intraoperative quantification of tissue perfusion^6–8^. HSI detects the relative reflectance of light with a wavelength comprised between 500 and 1000 nm, allowing the quantification of organic compounds, such as oxygenated and deoxygenated haemoglobin^9^. The application of HSI has recently gained importance for its non-invasiveness and the accuracy of oxygen quantification at different depths^10^. For that reason, HSI is a promising technology as it allows for the intraoperative quantification and spatial visualisation of hepatic oxygenation and to discriminate among different types of liver ischaemia.

We present a comparative preclinical study of HSI-based liver oxygenation quantification of porcine liver in an experimental model of total vascular inflow occlusion (VIO) versus hepatic artery occlusion (HAO). Optical properties extracted with HSI have been correlated with biological markers to predict the level of ischaemia. The HSI system used in this study provides presets for the quantification of the relative oxygen saturation (StO_2_%) at a depth up to ~ 1 mm and of the near-infrared (NIR) spectrum at a depth of up to 3–5mm^10^. Water distribution was assessed with the tissue water index (TWI), which quantifies tissue water content^10^.

## Results

### Spectral profile extraction

**Figure 1:**
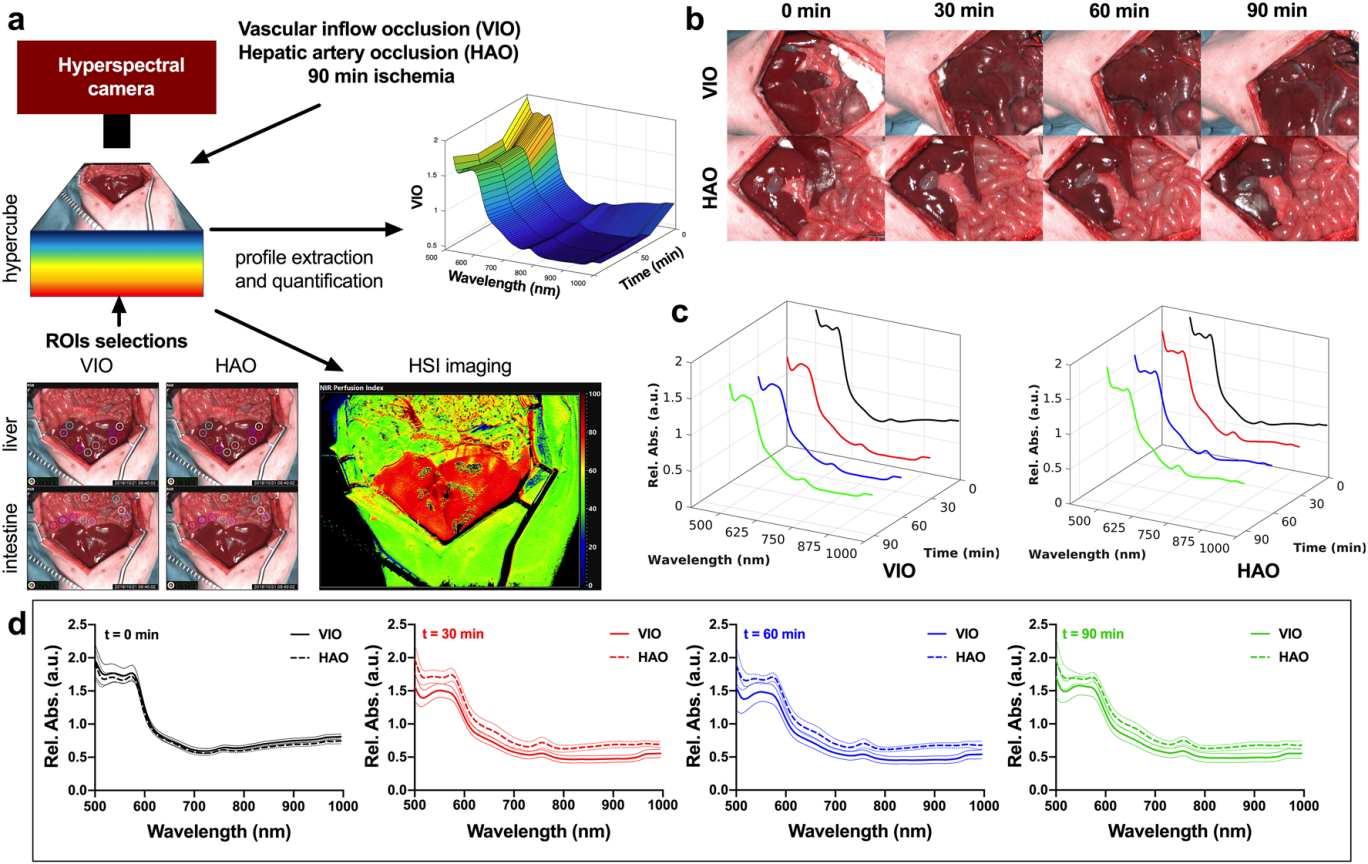
Experimental workflow. (**a**) Hyperspectral imaging was performed 3 times in 30-min intervals for a total period of 90 min in both VIO and HAO models. The images and spectra of 10 ROIs were extracted from the hypercubes and analysed. (**b**) RGB images of liver before ligation and during the ischemic phase, where no major differences were found. (**c**) Spectral profiles of both ischaemia types at different times showed that VIO control was overall higher as compared to the ischaemic phase. This was not the case for the HAO spectra. **(d)** Profiles comparison at each timepoint of VIO and HAO. Spectra extraction was performed with the TIVITA hyperspectral camera (Diaspective Vision GmbH, Germany) from 500 to 1000 nm with a resolution of 5 nm obtaining 250 bands per each image.

Hyperspectral images were collected during and before the ischaemic phase, following the selection of ten regions of interest (ROIs) in the intestine and in the liver, which allowed for the extraction of spectral profiles (hypercubes). Images and spectral profiles were extracted from the hypercubes of the ROIs (Fig. 1a). RGB images showed a slight colour difference during the ischaemic phase when compared to the control (Fig. 1b). The overall spectra of VIO showed a lower relative absorbance during the ischaemic phase when compared to the control and to all HAO timepoints (Fig. 1c,d).

### Liver hyperspectral imaging

**Figure 2:**
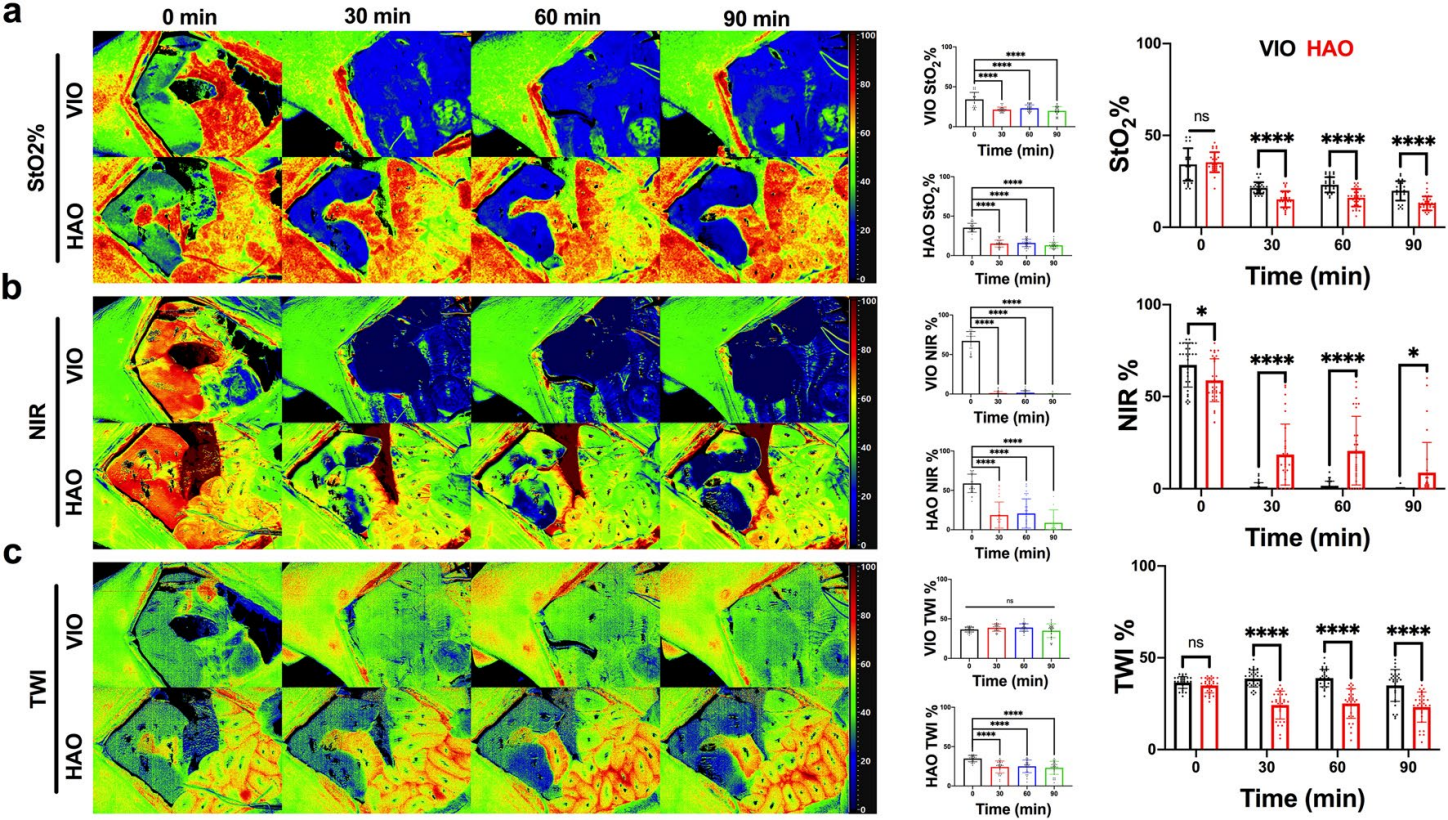
Liver hyperspectral imaging. (**a–c**) StO_2_%, NIR and TWI imaging and quantification. From left to right are displayed hyperspectral images of the two ischaemic models, the quantitative analysis of each model, and the comparison between VIO and HAO. All preset parameter indexes could distinguish different types of ischaemia at each timepoint. The images were quantified and finally compared with one-way and two-way ANOVA. Data are presented as mean ± s.d. and compared to the control, ns p > 0.05, *p ≤ 0.05, **p ≤ 0.01, ***p ≤ 0.001, ****p ≤ 0.0001. N = 60 (ROIs in 6 pigs).

The StO_2_% index showed a significant decrease during the ischaemic phase in both models (p < 0.001 for 30, 60 and 90 min in VIO and HAO) (Fig. 2a). When VIO and HAO were compared, StO_2_% in HAO was significantly lower (p < 0.0001, for 30, 60, and 90 min). The NIR index showed a visible change in VIO and HAO, similar to StO_2_%. However, it could detect a small difference also in the controls (p = 0.0335, p < 0.0001, p < 0.0001, p = 0.0286, for 0, 30, 60, and 90 min respectively). Additionally, NIR in HAO showed a localised area of ischaemia which increased in surface area over time (Fig. 2b). TWI did not show any significant difference in VIO ischaemia when compared to the control but showed a significant difference in HAO (p < 0.0001 for 30, 60, and 90 min). The HAO TW index was significantly lower when compared to VIO (p < 0.0001 for 30, 60 and 90 min) (Fig. 2c).

### Blood gas analysis

**Figure 3:**
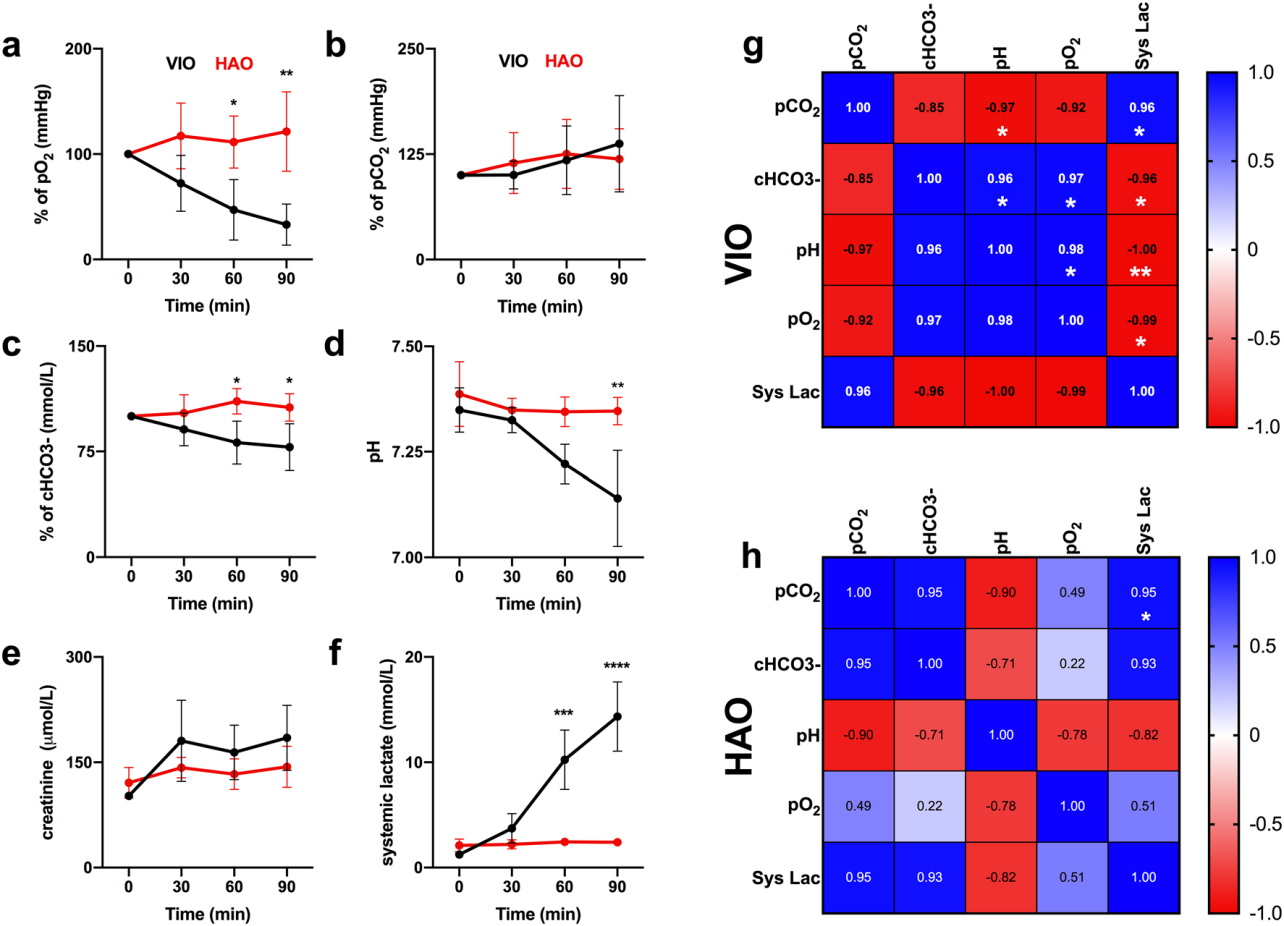
Monitoring of vital parameters with blood gas analysis (BGA). (**a–c**) pO_2_, pCO_2_, and cHCO_3_- were normalised with the control. (**d**) The pH was used to monitor the systemic acidosis. (**e**) Kidney function was monitored with the creatinine level. (**f**) Systemic lactate. (**g**) VIO correlation matrix of vital parameters. (**h**) HAO correlation matrix of vital parameters. Systemic lactates correlated better with pO2 in both VIO and HAO. Data are presented as mean ± s.d. and compared with one-way ANOVA to the control, ns p > 0.05, *p ≤ 0.05, **p ≤ 0.01, ***p ≤ 0.001, ****p ≤ 0.0001. N = 6 (pigs).

Partial oxygen pressure (pO_2_) decreased significantly over the ischaemic phase in VIO when compared to HAO after 60 min (p = 0.232), reaching a maximum spread after 90 min (p = 0.0019) (Fig. 3a). Partial carbon dioxide (pCO_2_) showed an overall non-significant increase in both VIO and HAO (Fig. 3b). There was a significant decrease in cHCO3-% after 60 min in VIO as compared to HAO (p = 0.0209) (Fig. 3c). The pH remained constant in HAO presenting a non-significant decrease after 30 min of ischaemia, which decreased significantly in VIO after 90 min (p = 0.0143) (Fig. 3d). Creatinine showed a slight increase, which was not statistically significant in VIO and HAO (Fig. 3e). Systemic lactate levels showed a dramatic increase in VIO, which was significant after 60 min, reaching a maximum spread at 90 min as compared to HAO (p < 0.0001) (Fig. 3f). The correlation matrix of VIO vital parameters showed that the pH was negatively correlated with pCO_2_ (r = − 0.97, p = 0.034) and positively with cHCO3- (r = 0.96, p = 0.041). In addition, it showed that pO_2_ was positively correlated with cHCO3- (r = 0.97, p = 0.034) and the pH (r = 0.98, p = 0.019). Systemic lactate was also found positively correlated with pCO_2_ (r = 0.96, p = 0.042) and negatively correlated with cHCO3-(r = -0.96, p = 0.035), the pH (r = − 1.00, p = 0.001), and pO_2_ (r = − 0.99, p = 0.011) (Fig. 3g). Finally, a positive correlation was found in systemic lactate levels with pCO_2_ in HAO (r = 0.95, p = 0.049) (Fig. 3h).

### Liver damage assessment

**Figure 4:**
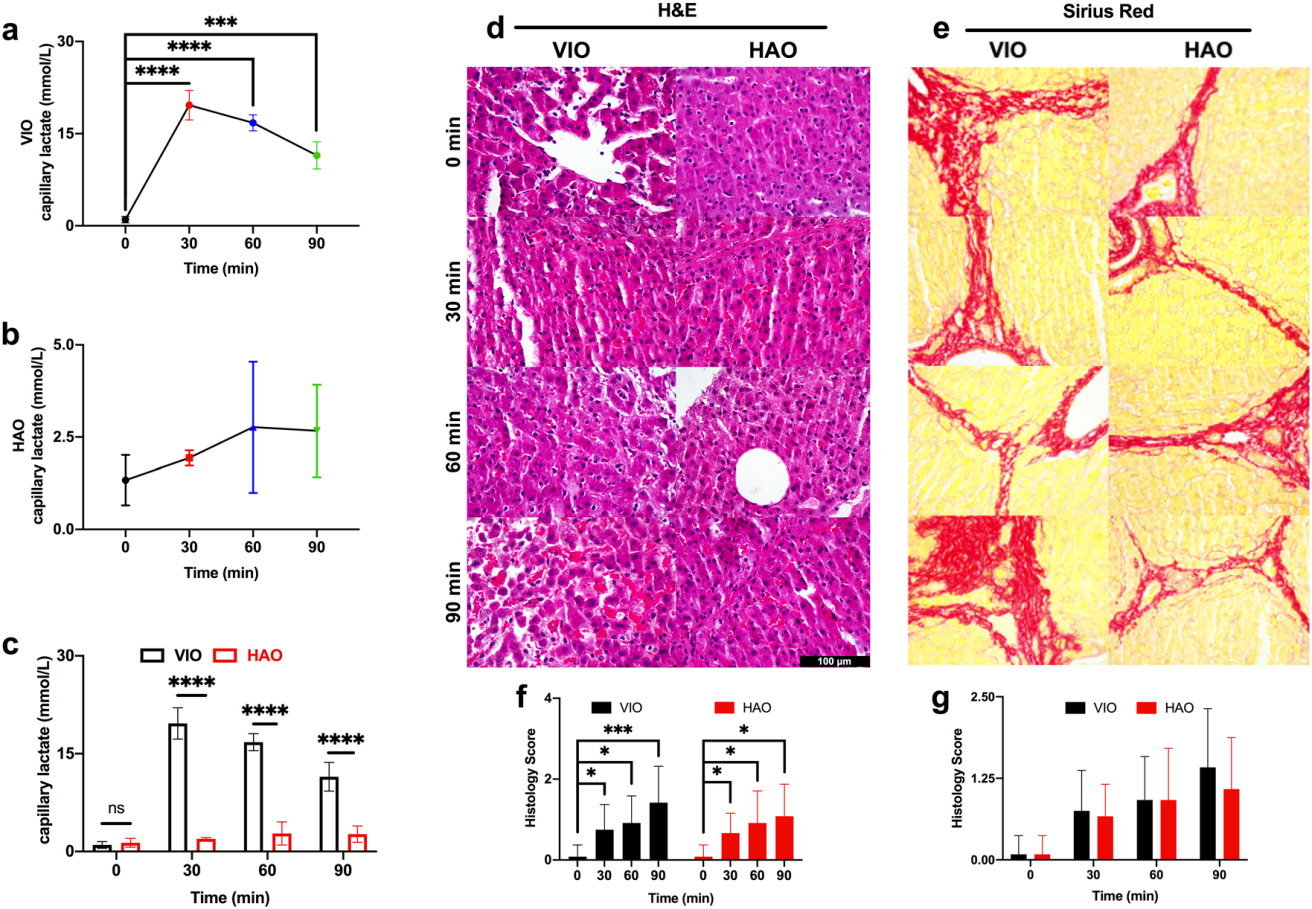
Liver biological analysis. (**a, b**) VIO and HAO capillary lactate. (**c**) Capillary lactate comparison between VIO and HAO. (**d**) H&E showed a gradual congestion increase in both ischaemic phases. (**e**) Sirius red did not show any change in the collagen structure which could affect HSI. (**f**) The histology score showed a significant increase in liver damage although (**g**) no significant difference was found between the two types of ischaemic models. Data are presented as mean ± s.d. and compared with one-way and two-way ANOVA to the control, ns p > 0.05, *p ≤ 0.05, **p ≤ 0.01, ***p ≤ 0.001, ****p ≤ 0.0001. N = 6 (pigs).

Capillary lactate sampled on the liver surface showed a significant increase in VIO after 30 min (p < 0.0001) followed by a decrease, while HAO showed no significant change (Fig. 4a,b). In addition, the difference between the two techniques was statistically different (p < 0.0001) for 30, 60, and 90 min (Fig. 4c). In VIO, the overall damage was represented by steatosis with hepatocyte atrophy and sinusoidal congestion. Similarly to HAO, necrosis was not the main parameter as compared to microvesicular steatosis or to congestion and atrophy. Pale staining was present in both ischaemic types from 30 min with an increasing trend up to 90 min. HAO was characterised by a lobular apoptosis, cholestasis, and neutrophils infiltration. Additionally, after 90 min, a strong congestion was found with a micro-haemorrhage close to the central vein and a micro-vacuolisation of hepatocytes cytoplasm (Fig. 4d). Sirius red staining did not show any major alteration in the collagen structure which could affect the hyperspectral signal in the control and the ischaemic phase (Fig. 4e). Overall, a significant gradual increase of histology score was found in both ischaemic models. Finally, there was no significant difference between the two histology scores in the two models for all timepoints (Fig. 4f,g).

### Intestine HSI imaging quantification

**Figure 5:**
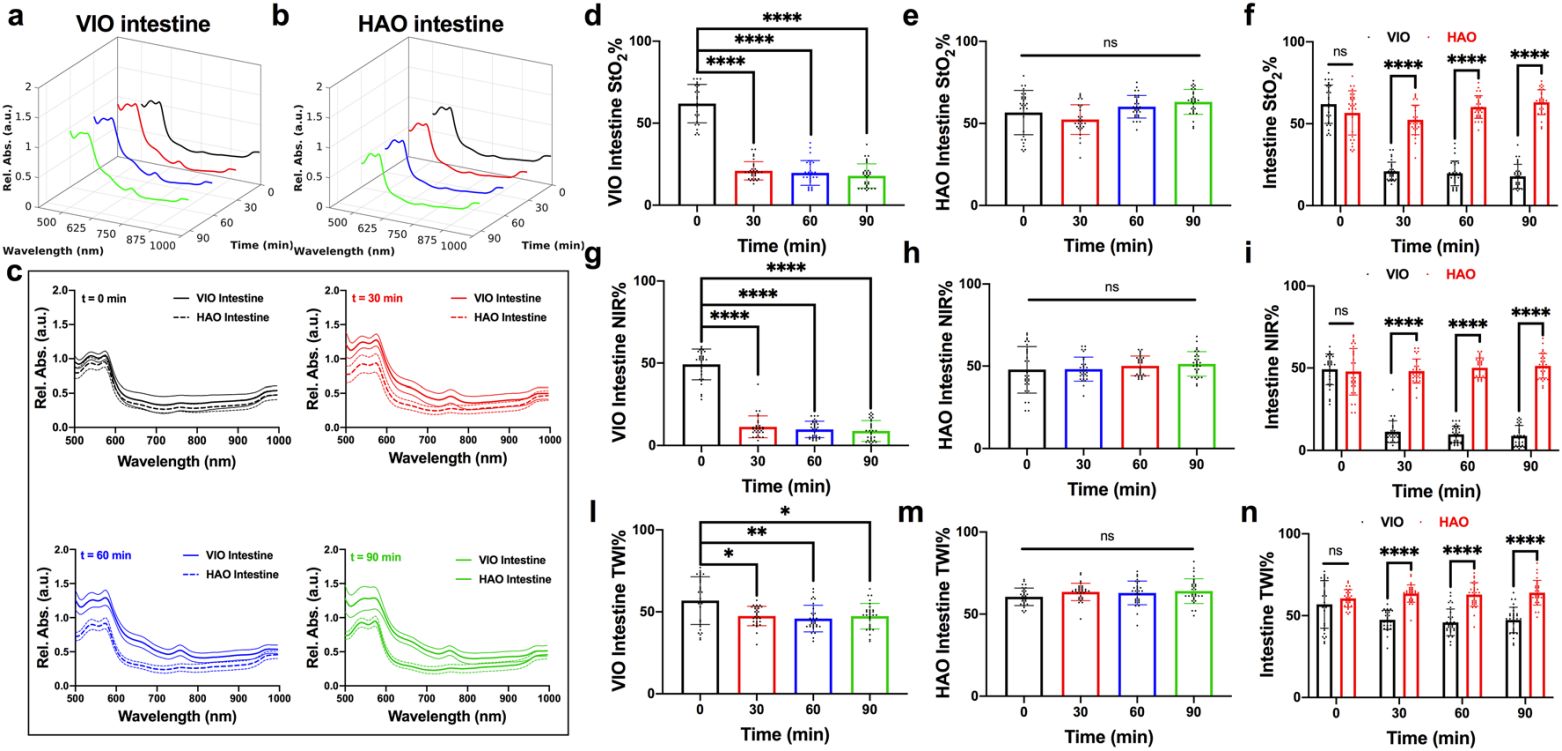
Intestine HSI imaging quantification. (**a, b**) Intestine spectra profile at different timepoints. (**c**) Intestine spectra comparison at every timepoint between VIO and HAO. (**d–f**) VIO and HAO StO_2_% quantitative comparison. (**g–i**) VIO and HAO NIR quantitative comparison (**l–n**) VIO and HAO TWI quantitative comparison. Data are presented as mean ± s.d. and compared with one-way and two-way ANOVA to the control, ns p > 0.05, *p ≤ 0.05, **p ≤ 0.01, ***p ≤ 0.001, ****p ≤ 0.0001. N = 60 (ROIs in 6 pigs).

Intestinal congestion, if used as a cross-validation test, is potentially an additional element for an automatic diagnosis. We subsequently analysed this parameter to confirm the experimental model. The spectral profile of the intestine showed an increase in relative absorbance in VIO but not in HAO during the ischaemic phase (Fig. 5a,b). The difference between the two spectra at every timepoint was maintained during the whole procedure (Fig. 5c). The VIO StO_2_% showed a significant decrease in oxygenation when compared to the control, which was not observed for HAO (Fig. 5d,e). Intestinal comparison of StO_2_% showed a significant difference between VIO and HAO (p < 0.0001 for 30, 60, and 90 min) (Fig. 5f). Similar results were observed with NIR and TWI (Fig. 5g, h, i, l, m, n).

### Correlation

**Figure 6:**
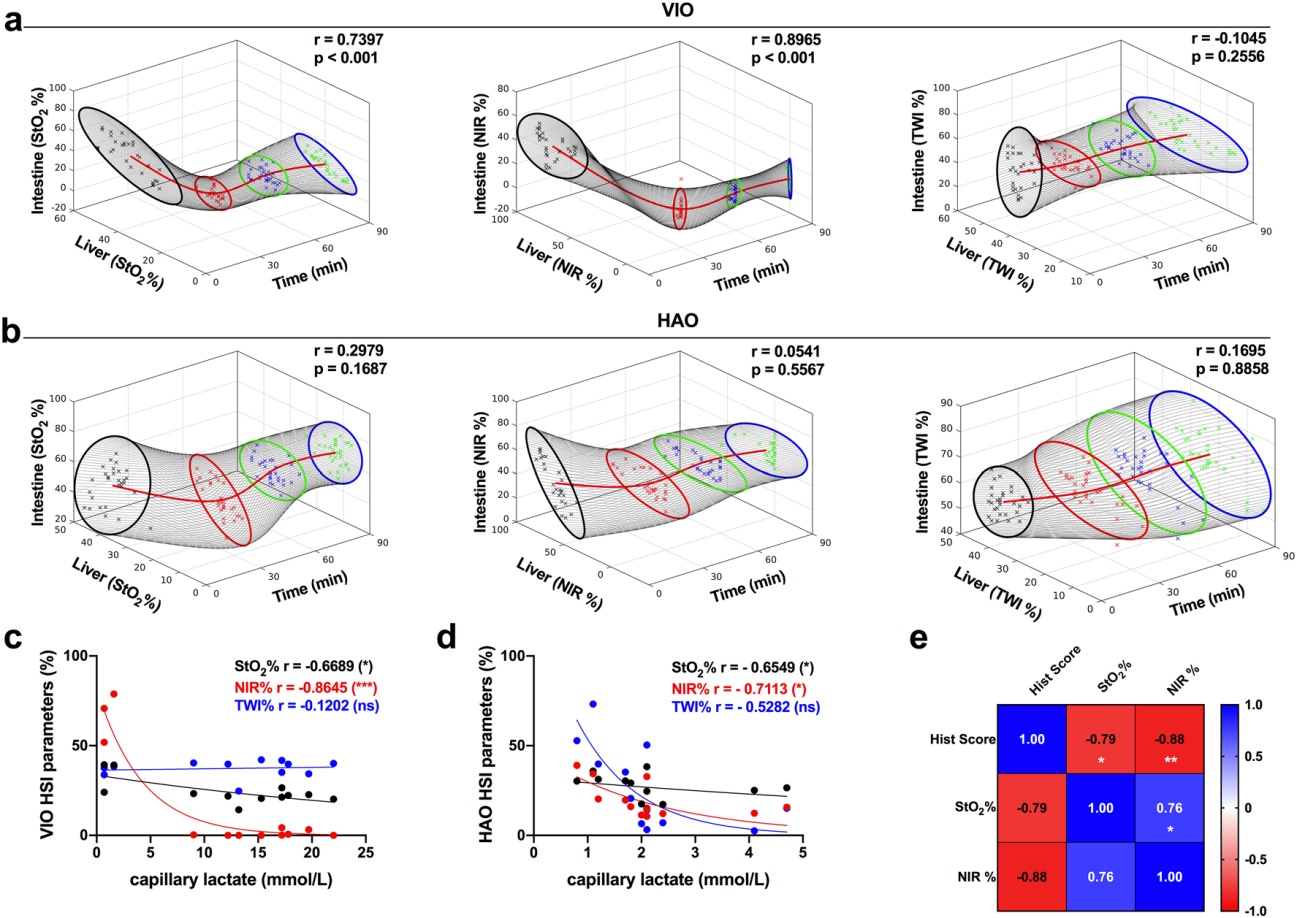
Correlation analysis. (**a**) VIO HSI parameter correlation of liver and intestine over time (**b**) HAO HSI parameter correlation of liver and intestine over time. (**c, d**) Capillary lactate and HSI parameters correlation in VIO. N = 120 (6 pigs, 10 ROIs). (**e**) Comparison between histology score, StO_2_%, and NIR. Data are correlated with Pearson’s and Spearman’s analysis, ns p > 0.05, *p ≤ 0.05, **p ≤ 0.01, ***p ≤ 0.001, ****p ≤ 0.0001. Visualisations of the correlation between TWI, StO_2_%, and NIR over time. At each timepoint, a scatter plot is visualised, with a best-fitting ellipse at the 95% confidence interval using Principal Component Analysis. N = 24 (6 pigs).

StO_2_% and NIR showed a significant correlation between liver and intestine in VIO, except for TWI (Fig. 6a). No correlation was found in HAO in any of the HSI parameter indexes (Fig. 6b). A stronger correlation between NIR and capillary lactate was found in VIO and HAO when compared to StO_2_% and TWI (p = 0.0003 and p = 0.0120 respectively) (Figs. 6c,d). Finally, the histopathological score showed a significant negative correlation between StO_2_% and NIR (r = − 0.79, p = 0.018 and r = − 0.88, p = 0.004 respectively). A positive correlation was also found between StO_2_% and NIR (r = 0.76, p = 0.029) (Fig. 6e).

## Discussion

HSI is a non-invasive and easy-to-use imaging technology recently applied to the medical field, providing a contrast-free biochemical analysis of tissues based on optical endogenous properties^11^. In addition, HSI has been successfully applied as a tool to discriminate healthy from pathological tissue and for the quantification of organ perfusion level in both preclinical and clinical settings^12–14^. The ability of the HSI system to evaluate the liver oxygenation was already assessed and applied by our group in an intraoperative setting to create hyperspectral enhanced reality (HYPER) in liver anatomical resection^15–17^. However, doubts remained as to whether or not the system could discriminate different types of hepatic vascular occlusions. We therefore tested the system to explore this additional feature that we considered important. This is especially true in liver transplantation for dreaded vascular complications such as hepatic artery thrombosis which can lead to up to ~ 60% of retransplantation and a ~ 50% of mortality rates^18^.

Currently, US is the standard and approved clinical intraoperative imaging tool for liver perfusion monitoring. However, US requires specific training and its interpretation may vary among the authors. Additionally, US does not provide a global map of liver oxygenation intraoperatively, while HSI provides a spatial resolution which can help the operator to localize the ischemia.

To the best of our knowledge we found some experimental methods to monitor liver ischemia. All of these promising techniques such as CO_2_ sensor, indocyanine green (ICG) and near infrared spectroscope, presented drawbacks such as invasiveness, the lack of spatial and informative quantification of ischaemia, the non-intraoperative applicability^19–23^. These major drawbacks make it difficult to transfer these methodologies to the clinical practice. For instance, ICG recently gained importance as intraoperative imaging assessment for liver perfusion^21,24^. Although this technique showed positive results, it is still an invasive methodology due to the need for an injection of an exogenous molecule, which requires time for the clearance. Additionally, some patients are allergic to the iodine and its application for imaging purposes has not been approved. The HSI technology overcomes these problems intraoperatively thanks to its non-invasiveness and its standardised perfusion assessment which is not operator dependent. Additionally, HSI provide for an optical quantification of endogenous molecules for which a labelled dye injection is not needed. In this study, we tested the potential of HSI ability to detect possible complications in liver surgery associated with oxygen impairment intraoperatively. HSI could precisely predict the level of capillary lactate concentration from changes in optical properties. These results are useful to create an automatic diagnostic system based on optical imaging, which may predict liver graft dysfunction. In our experiments, the camera facilitated the automatic quantification of three parameters which can otherwise be extracted from the hypercube manually^10^. Bile has a spectral profile which could interfere with the StO_2_% and NIR parameters^25^. For that reason, the bile duct was left out of the Pringle’s manoeuvre to prevent any bile obstruction which could gradually impair the HSI signal. NIR images showed the ability to localise and quantify the level of ischaemia in the HAO manoeuvre (Fig. 2b). This is probably because NIR wavelengths have deeper tissue penetration as compared to StO_2_%^10^. This aspect, together with the need to implement the bile signal, may make StO_2_% images less informative. However, the lack of a corrective algorithm of the bile spectra profile, limits the applicability of such technology in scenarios on which the bile duct is occluded for long time. Nonetheless, we consider this limit possible to overtake by the creation of an ad hoc plug-in of the proprietary software.

TWI did not reveal any changes in VIO, probably due to the full clamp of the vascular inflow which blocked the circulation, as confirmed by the sinusoidal congestion (Fig. 3d). For VIO, TWI did not detect any statistical difference. Given the total inflow occlusion, the blood flow was almost completely stopped and the water contained in tissues and blood stayed inside the organ. The overall significant difference detected by TWI in the HAO ischaemic phase was then likely due to a relative reduction in blood flow. When the hepatic artery was occluded in HAO, the portal blood flow did not buffer the arterial flow. Indeed, differently from the occlusion of the portal vein, there is no such compensatory mechanism as the HABR effect^26^. Additionally, the post-sinusoidal pressure was maintained via systemic circulation throughout the inferior vena cava^27^. Consequently, TWI in the liver may be mainly influenced by blood circulation in the liver.

Vital parameters confirmed that the experimental workflow was coherent with the pathophysiology of both ischaemia types. Lactate was the blood marker which correlated with pCO_2_% in VIO and HAO. It has been previously used as the main marker to assess correlation between optical and biological properties^28^. Capillary lactates in VIO increased dramatically after 30 min and started to decrease gradually over time. This was likely due to their distribution into the systemic circulation via the inferior vena cava. The ischemic process could have been non-homogeneous, impairing histology assessment. Bigger biopsies could partially solve this problem but would affect microcirculation, creating a bias in the study. We quantified the following 4 parameters: coagulative necrosis, micro-vacuolisation of the cytoplasm, pale staining, and sinusoidal congestion. A damage increase was characterised by micro-vacuolisation and congestions which appear statistically significant. These alterations appeared more significant after 60 min. Sinusoidal congestion was the most interesting histopathological parameter. This is fundamental in liver vascular thrombosis due to the slowdown in the hematic flow with a dramatic reduction of the sinusoidal pressure which reduces the chance of the blood to wash out into the central vein. Due to its ability to quantify the light scattering, the HSI signal also depends on the tissue texture. This property has been exploited for diagnostic purposes^29^. In order to rule out any possible bias given by any potential alteration in liver microarchitecture, Sirius red staining was performed, showing no difference in collagen structure in the control and ischaemic phases. In VIO, the blood flow from the portal vein was interrupted creating a congestion in the intestine, while the blood flowed through the liver in HAO. This was coherently differentiated in HSI as shown in Fig. 5. This was also shown in Fig. 6, where there was no correlation in HAO between the liver and the intestine. However, there was a high correlation in VIO. The overall correlation between the HSI parameters and capillary lactates showed that NIR is a better parameter to detect changes in liver oxygenation and, as a result, in liver hypoxia (Fig. 6c,d). This is due to the similar depth between NIR imaging and blood sampling (~ 3–5 mm) while the StO_2_% is more superficial at ~ 1 mm. At that depth, the blood is mainly characterised by arterial circulation. Consequently, the overall evaluation of liver hypoperfusion would be impaired in HAO^30^. Therefore, StO_2_% would be influenced by a higher sensitivity with a dramatic loss of signal as compared to VIO (Fig. 2a,b). Finally, the histopathological score showed a high correlation between StO_2_% and NIR, confirming together with the capillary lactate that HSI parameters can predict the overall level of ischaemia. It is still unclear if HSI can distinguish different times of liver ischaemia within the same ischaemic model. To achieve this goal, a possible next step would be to apply an artificial intelligence-based analysis of the whole spectra associated with the automatic recognition of liver tissue to distinguish VIO, HAO, portal vein occlusion (PVO), and bile duct occlusion (BDO) at different timepoints. The overall correlation with biological data confirmed that HSI is a suitable tool for intraoperative diagnosis and as a result, it merits further investigation for future preclinical and clinical studies using the abovementioned models. Although HSI could discriminate and quantify two different models of hepatic ischaemia, its preset parameters could not discriminate ischaemic time points. In fact, both in liver and intestine, there was no statistical difference among the ischemic time points regarding all parameters. The additional study of time is crucial for an early intervention to prevent the reperfusion damage. Consequently, a future study on the discrimination of the ischemic time points is more than necessary.

## Methods

### Study design

The primary aim of this study was to evaluate the accuracy of intraoperative HSI in the quantification of liver oxygenation in two models of hepatic ischaemia (VIO and HAO). In order to achieve this goal, HSI was used to extract a relative absorbance between 500 and 1000 nm from hyperspectral images and to quantify the relative absorbance^10^. The optical data were compared to capillary lactates to measure the correlation between optical and biological properties. Histopathological characterisation and quantification were performed to confirm the experimental workflow and evaluation. The data were collected before ligation and every 30 min after ligation for a total duration of 90 min.

### Animals

This study is part of the ELIOS project (Endoscopic Luminescent Imaging for Oncology Surgery). It was approved by the local Ethical Committee on Animal Experimentation (ICOMETH No. 38.2016.01.085), as well as by the French Ministry of Superior Education and Research (MESR) (APAFIS#8721-2017013010316298-v2). All animals were managed according to French laws for animal use and care and the directives of the European Community Council (2010/63/EU) and ARRIVE guidelines^31^. Six adult male swine (*Sus scrofa ssp. domesticus*, mean weight: 29.4 ± 4.8 kg) were housed and acclimatised for 48 h in an enriched environment, with constant humidity and temperature conditions. A fasting period was held for 24 h before surgery, with ad libitum access to water. Stress was reduced by means of sedation (zolazepam + tiletamine 10 mg/kg IM) 30 min before the procedure and respecting circadian cycles of light-darkness. Propofol (3 mg/kg) was injected intravenously (18 gauge IV catheter in ear vein) and maintained with rocuronium 0.8 mg/kg along with inhaled isoflurane 2%. Animals were euthanised with a lethal dose of pentobarbital (40 mg/kg) at the end of the procedure.

### Sample size calculation

Correlation between capillary lactate and StO_2_% HSI preset parameters was used as a primary outcome to compute sample size. The calculation was based on previous publications on bowel ischaemia, which showed a ρ correlation coefficient of − 0.7^16,32^. The required paired values were 4, considering α = 0.05 with a power (1 − β) = 0.9. In the present study, 120 paired StO_2_% lactate values were obtained in six pigs.

### Surgical procedure

After a midline laparotomy and hepatic pedicle dissection, two different vascular inflow occlusions were performed: (1) total occlusion of the vascular inflow (VIO), and (2) hepatic artery isolation and occlusion (HAO). Both manoeuvres were performed for 90 min, which has been shown to be sufficient to induce liver damage in pig models^33^. The bile duct was isolated to prevent its ligation.

### Hyperspectral imaging

Hyperspectral camera can acquire the spatial 2D image with a third dimension represented by spectroscopic information in a determined wavelength field. In this experimental study, we used a CMOS pushbroom scanning hyperspectral camera (TIVITA, Diaspective Vision GmbH, Germany) to generate hyperspectral images. The distance between the camera and the organ was 40 cm. The distance was monitored by a distance sensor (Bluefruit Feather nRF52832 with Adafruit VL53LOx device) during the whole procedure. The images were acquired using 20 W Osram Halospot 70 Halogen lamp (6 ×) allowing a range of spectra of 500 to 1000 nm. The HSI system took ~ 6 s to perform the imaging. The cube data was transferred to a PC where it was processed creating pseudo-colour images. Data of the cube has dimension of 640 × 480 × 100 (px × px × wavelength). Relative reflectance was extracted within the wavelength range with a gap of 5 nm for a total number of 250 spectral samples. The 3D cube extracted contained the spatial data with the relative reflectance ($\frac{I}{{I}_{0}}$) for each pixel that was converted in relative absorbance via the system through the equation: $A=-\mathrm{ln}\left(\frac{I}{{I}_{0}}\right).$ Quantitative analysis of StO_2_%, NIR, and TWI were performed intraoperatively using the TIVITA software^34^. HSI was performed before ligation (t = 0 min), and every 30 min for a period of 90 min (t = 30, 60, 90 min) of ischaemia. Quantitative analysis was performed obtaining the HSI parameters by selecting 10 ROIs in the liver and in the intestine at each timepoint. Average NIR, StO_2_%, and TWI preset parameters were computed within each ROI using the TIVITA software.

### Biological analysis and quantification

Creatinine, pO_2_, pCO_2_, cHCO3-, pH, and systemic lactate levels were analysed to monitor global kidney injury, overall acidosis, and lactate production. Blood was sampled through a catheter placed inside the jugular vein (18 gauge IV catheter) under ultrasound guidance and analysed with the epoc Blood Analysis System (Siemens Healthineers).

Capillary lactate levels were measured using a strip-based portable lactate analyser (EDGE, ApexBio, Taipei, Taiwan; error margin 0.35 mmol/L), from blood samples obtained from the liver surface by puncturing Glisson’s capsule. The order of sampling from liver segments was randomised. HSI preset parameters and capillary lactates were correlated to assess the relationship between optical properties and the actual hypoxic metabolism.

### Histology

Liver biopsies were taken randomly from posterior segments. Formalin-fixed paraffin-embedded (FFPE) sections of 5 μm were stained using Harris Hematoxylin formula (Leica Biosystems) and Picro Sirius Red Stain (Sigma-Aldrich) according to the manufacturers’ instructions. The histopathological score was assigned by a pathologist who was blinded to the experimental conditions, creating a scoring chart similar to Suzuki’s^35^. The score was based on the following variables: (1) cell necrosis, (2) vacuolisation, (3) pale staining, (4) congestion. The score was evaluated using the following scale: (0) none, (1) mild, (2) moderate, and (3) severe.

### Statistical analysis

Statistics were performed with GraphPad 8.3 (Prism, GraphPad Software, San Diego, CA, USA). Pearson’s and Spearman’s rho were calculated to correlate local lactates with HSI parameters. One-way mixed-effect model ANOVA with Dunnett’s multiple comparisons, Kruskal–Wallis with Dunnett’s multiple comparison test and Brown-Forsythe and Welch ANOVA test with Dunnett’s multiple comparison test were performed accordingly with the assumption of the data distribution. Two-way ANOVA with Sidak’s multiple comparisons were performed to calculate differences in continuous variables for parametric tests. Pearson’s and Spearman’s correlations were applied depending on the assumptions of data distribution. A two-tailed analysis with *p value* < 0.05 was considered statistically significant. Three-dimensional scatter plots were performed with MATLAB 2014a.
